# Signaling pathway mechanisms of neurological diseases induced by G protein‐coupled receptor 39

**DOI:** 10.1111/cns.14174

**Published:** 2023-03-21

**Authors:** Bin Cao, Jue Wang, Juan Feng

**Affiliations:** ^1^ Department of Neurology Shengjing Hospital of China Medical University Shenyang China

**Keywords:** GPR39, GPR39 agonist, GPR39 signaling, zinc

## Abstract

**Background:**

G protein‐coupled receptor 39 (GPR39) is a transmembrane zinc receptor with two splice variants, which belongs to the G‐protein‐coupled receptor growth hormone‐releasing peptide family. Its expression is induced by zinc, which activates GPR39, and its activation mediates cell proliferation, ion homeostasis, and anti‐inflammatory, antioxidant, and other pathophysiological effects via different signaling pathways.

**Aims:**

The article reviews the latest literature in this field. In particular, the role of GPR39 in nervous system is discussed.

**Materials and methods:**

GPR39 can be a promising target in neurological diseases for targeted therapy, which will help doctors overcome the associated problems.

**Discussion:**

GPR39 is expressed in vivo at several sites. Increasing evidence suggests that GPR39 plays an important role as a neuroprotective agent in vivo and regulates various neurological functions, including neurodegeneration, neuroelectrophysiology, and neurovascular homeostasis.

**Conclusion:**

This review aims to provide an overview of the functions, signal transduction pathways, and pathophysiological role of GPR39 in neurological diseases and summarize the GPR39 agonists that have been identified in the recent years.

## Graphical abstract

The expression of G protein‐coupled receptor 39 (GPR39) is induced by zinc, which activates GPR39 and later mediates cell proliferation, ion homeostasis maintenance, anti‐inflammatory, antioxidant, and other pathophysiological effects through different signaling pathways. It plays an important neuroprotective role in neurodegeneration, neuroelectrophysiology, and neurovascular homeostasis.

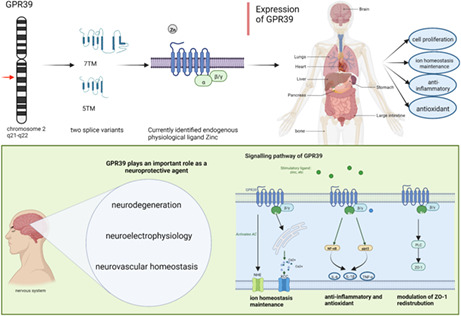

## 1 INTRODUCTION

G protein‐coupled receptor 39 (GPR39) is an orphan G protein‐coupled receptor (GPCR) with constitutive activity, which was discovered by McKee et al.^1^ It belongs to the growth hormone‐releasing peptide receptor family, which consists of peptide hormones, neuropeptide‐activated growth hormone‐releasing peptide, neurohypocretin, and gastrin receptors.^2^ However, the natural ligand of GPR39 is unknown. Reports have suggested that GPR39 is a ligand‐free orphan receptor that can activate downstream signaling pathways, including oligomerization.^3^ Moreover, obestatin is considered as a ghrelin‐related peptide derived from post‐translational processing of the preproghrelin gene and as an endogenous agonist of GPR39; however, this conclusion has been refuted.^2^, ^4^ After extensive studies, Zn ions were found to be endogenous ligands for GPR39, and Zn^2+^ was observed in a serum screen for GPR39 agonists.^5^ GPR39 activation requires transient changes in extracellular Zn^2+^, and an exogenous application of Zn^2+^ may trigger ZnR/GPR39 activation. However, endogenous sources of vesicular Zn^2+^, namely, neuronal and salivary gland vesicles, pancreatic enzymes, or panniculocytes in the intestine, may serve as physiological triggers for ZnR/GPR39 activation. Zn^2+^ is released from damaged cells, and it activates ZnR/GPR39 signaling. Based on previous studies, ZnR/GPR39 activity could enhance neuronal inhibitory tone, and Zn deficiency is associated with epilepsy and seizures, indicating the important physiological role of ZnR/GPR39.^6^, ^7^, ^8^, ^9^

Human GPR39 is a single‐copy gene localized on chromosome 2 q21‐q22, and it encodes the 453 amino acid GPR39 protein,^1^ which is primarily expressed in the pancreas, liver, adipose tissue, and gastrointestinal tract. Recently, GPR39 was found to be expressed in mouse oviduct epithelium, with weak expression in the jugular and isthmus.^10^ GPR39 is highly expressed in several regions of the central nervous system (CNS), such as the amygdala and hippocampus, and it is involved in many important metabolic and endocrine functions. GPR39 plays a role in regulating a variety of physiological functions, including cell proliferation, differentiation, and survival. Moreover, it has been implicated in various diseases such as epilepsy, depression, dementia, cardiovascular disease, salivation, osteoporosis, inflammation, male infertility, and cancer.^11^, ^12^, ^13^, ^14^, ^15^ Based on recent studies, the addition of dietary Zn or a small‐molecule GPR39 agonist can effectively enhance thymic function in a mouse model of allogeneic hematopoietic cell transplantation (HCT). Thus, it may be an innovative approach for the treatment of HCT.^16^ Perkey et al. suggested that dying thymocytes release Zn^2+^ into the extracellular space, which activates endothelial cells via GPR39. GPR39 signaling triggers the release of BMP4 (a member of the bone morphogenetic protein family). BMP4 release maintains cortical and medullary thymic epithelial cell (cTEC and mTEC) regeneration and promotes ab initio T‐cell production.^17^ One study found that ZnR/GPR39 is expressed in the human submandibular gland and HSG cells.^18^ ZnR/GPR39 triggers the Zn^2+^‐induced translocation of aquaporin 5, an indicator of salivary gland secretion, in HSG cells by increasing Ca^2+^i.^18^ Some studies have also reported that GPR39 promotes skin healing.^19^ The activation of GPR39 shows beneficial effects on the body. Despite the limited specific regulatory compounds of GPR39, it is still expected to make a breakthrough in the experiment.

GPCRs, also known as seven‐transmembrane (7TM) receptors, are the largest superfamily of membrane receptors, which include >800 vertebrate transmembrane proteins that play important roles in physiology and other diseases. In numerous experiments, Zn plays an essential physiological role in vivo.^6^ As a highly specific ZnR, GPR39 is expressed in many tissues in vivo, which indicates that GPR39 may be involved in various pathophysiological roles in vivo. In the nervous system, previous results show that GPR39 has positive and beneficial neuroprotective effects on the nervous system. Future studies investigating whether GPR39 agonists can selectively activate GPR39‐dependent signaling pathways would provide new ideas and targets for the treatment of currently incurable neurological diseases. This review focuses on the mechanism of action of GPR39 in vivo and ex vivo, associated signaling pathways, and important roles of these factors in different neurological diseases and summarizes recent findings in GPR39 agonists.

## 2 COMPOSITION OF THE GPR39 RECEPTOR

GPR39 encodes two isoforms: GPR39‐1a and GPR39‐1b. GPR39‐1a is a full‐length 7TM receptor consisting of extracellular N‐terminal and transmembrane structures 1–7 (TM I‐VII; stable A domain and free B domain), with an intron in between. In addition, GPR39‐1b is a truncated 5th transmembrane isoform of GPR39, which consists of the extracellular N‐terminus and transmembrane structure domains 1–5 (TMI‐V; relatively stable A domain). Three exons of GPR39 overlap with an antisense gene known as LYPD1 (Ly‐6/PLAUR structural domain contains 1), and the LYPD1 antisense gene is highly expressed throughout the CNS.^20^, ^21^ A study on human GPR39 revealed that this gene consists of two exons separated by an intron of ~200 kb.^20^ This finding demonstrated that GPR39 may be similar to gastrin and growth hormone‐releasing peptide receptors and the possibility of two splice ligands. Moreover, GPR39‐1b can homodimerize, but it does not heterodimerize with GPR39‐1a.^21^ Yoshinobu et al. found that GPR39‐1b regulates food intake, and its expression may be the highest in the small intestine and stomach.^22^ GPR39‐1b is highly expressed in the cerebellum, pons, and other intracranial sites, such as the hypothalamus, cortex, and hippocampus. Similarly, GPR39‐1b forms a heterodimer with neurohypophyseal receptor 1 (NTSR1). Furthermore, neurohypophyseal receptor 2 (NTSR2) is a subtype of NTSR1, and its structural domain is similar to that of GPR39‐1b. NTSR2 is predominantly and intracranially expressed.^21^ However, full‐length GPR39‐1a is not significantly expressed in the CNS.^20^

In the early 20th century, Laura et al. demonstrated that GPR39 has two disulfide bonds with distinct functions in its extracellular structural domain. The conserved disulfide bond between Cys108 and Cys210 at the extracellular terminus of TM‐III is required for proper cell‐surface expression and agonist‐mediated activation. However, Cys11 in the N‐terminal structural domain is linked to Cys191, and it is not required for expression or receptor activation, but it is important for the inhibitory effect of ligand‐activated receptor signaling in the extracellular structural domain.^23^ The CWXP motifs Trp‐VI:13 and Phe‐V:13 are stable because of their aromatic interactions, and Trp‐VI:13 and Phe‐V:13 are important for constitutive activity. Both motifs are less effective for agonist‐induced efficacy, and they have no effect on agonist potency. Phe‐V:13 activates the metastable isomer, whereas Trp‐VI:13 changes by rotating to the ideal position and interacting with Phe‐V:13. This change is important for the overall conformational changes that occur in TM‐V and TM‐VI during receptor activation.^24^ This process also indicates the important molecular communication between TM‐VI and TM‐V during 7TM receptor activation.

GPR39‐1A was expressed selectively, whereas GPR39‐1B was expressed widely. Differences in expression patterns and structures are observed between the two splicing variants. In the CNS, the expression level of the two splicing variants is different.^20^ At present, no report has been found on the specific functional difference between them in the CNS, which may be due to the low expression level of GPR39‐1A, which cannot be detected. Thus, scholars focus on the pathophysiological effects and mechanisms of GPR39 activation.

## 3 GPR39‐RELATED PATHWAYS

### 3.1 GPR39 regulates downstream signaling pathways

**FIGURE 1:**
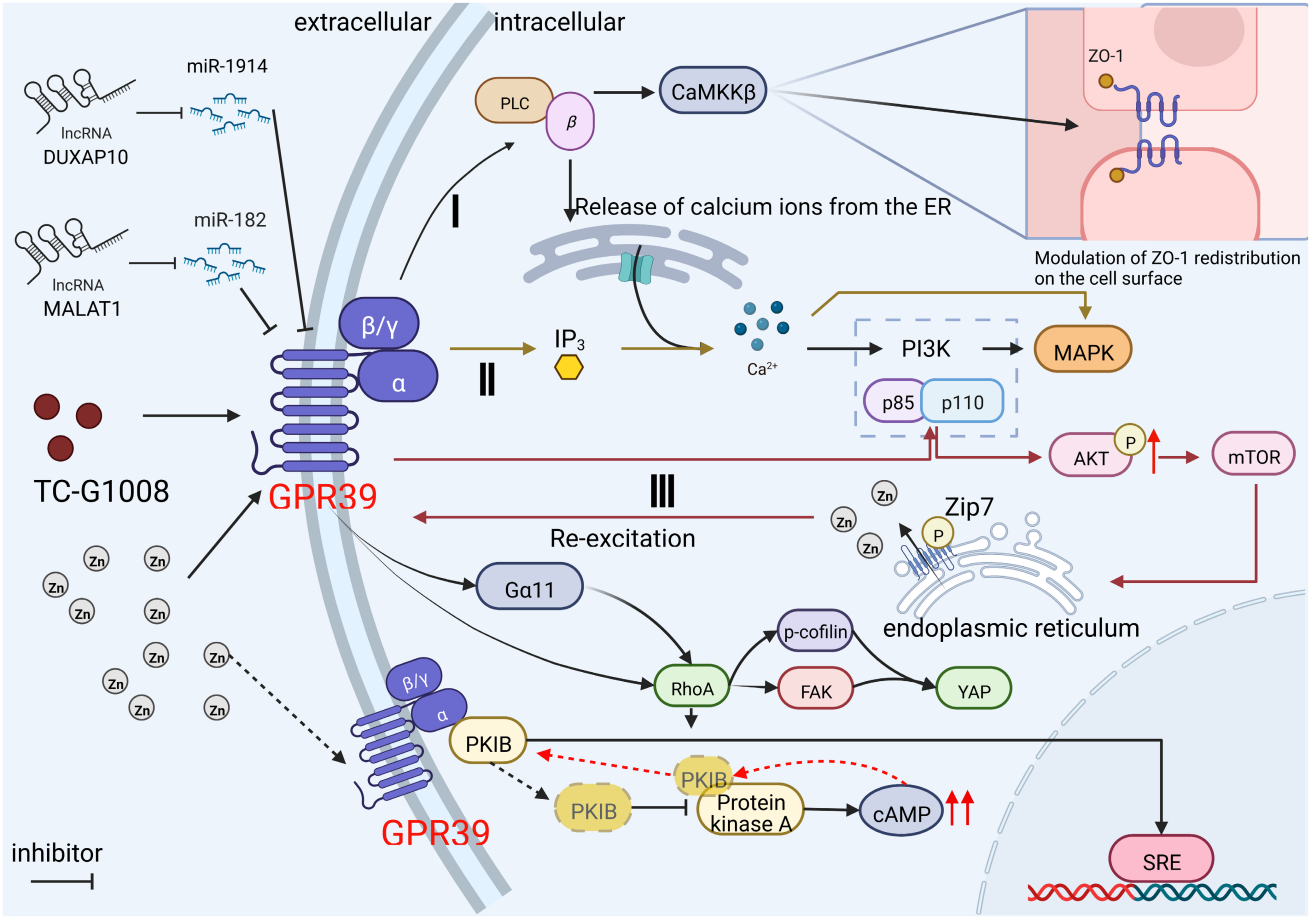
Two miRNAs, zinc ions, and specific agonist TC‐G1008 regulate the expression of GPR39. The figure includes downstream pathways I, II, and Gα11 for the classical Gαq‐like signaling pathway of GPR39, which can mediate the release of intracellular calcium ions and the transcription of SRE. Pathway III delays the activation of GPR39 by agonizing the PI3K/Akt/mTOR pathway. A specific modality of regulation occurs in concert with PKIB, which later inhibits PKA activity, thereby inducing the activation of cAMP. It can promote the dissociation of PKA and increase the binding of PKIB to GPR39, which also forms a negative feedback regulation.

After its discovery, GPR39 was thought to be an orphan receptor with no associated ligand activation, which could potentially activate downstream signaling pathways through dimerization or oligomerization, among other pathways.^3^ After relentless scholarly efforts, early studies exploring the ligands of GPR39 showed that the calcium‐mobilizing activity of Zn^2+^ was not eliminated by pertussis toxin but by the phospholipase C (PLC) inhibitor U73122. Therefore, the activity of GPR39 is mediated by the Gq alpha‐PLC pathway.^5^ Ongoing studies have demonstrated that GPR39 is highly selective for Zn and conserved across species. Zn is involved in a variety of intra‐ and extracellular reactions in vivo, and many of these reactions may be regulated by GPR39 (Figure 1). Zn^2+^ binds to ZnR/GPR39 through two histidine residues (His17 and His19) and one aspartate residue (Asp313).^23^ Zn is involved in various cellular reactions in vivo, some or all of which exert their biological effects via GPR39.

In the nervous system, the GPR39 agonist decreases the expression level of caspase3, caspase9, AIF, and BAX mRNA after high‐dose corticosterone treatment, increases the expression level of BCL‐2 mRNA, and protects HT‐22 cells.^25^ Researchers have observed that hippocampal CREB, BDNF, and TrkB are downregulated in GPR39 knockout mice. The results were the same in mice and rats, where the expression level of GPR39, CREB, BDNF, and TrkB was downregulated in the hippocampus of Zn‐deficient rats and mice. The antidepressant properties of TC‐G 1008 and Zn deficiency may activate the GPR39/CREB/BDNF/TrkB pathway in the hippocampus.^26^, ^27^ Moreover, the levels of GPR39 in the frontal cortex and hippocampus were significantly decreased in suicide victims upon autopsy.^24^ Reduced levels of CREB and BDNF proteins were found in the hippocampus of GPR39 KO mice, indicating that GPR39 is involved in the synthesis of CREB and BDNF.^28^

Homeostasis among glutamatergic, monoaminergic, and GABAergic agents can affect mood, and it may cause psychological disorders, such as anxiety and depression. The results indicate that inhibiting serotonergic, norepinephrine, or dopamine delivery by injection of pCPA or αMT can downregulate the expression level of GPR39 after 10 days, but it did not affect serum Zn levels. This result suggests the potential role of GPR39 in glutamatergic and monoaminergic neurotransmission.^29^

The role of GPR39 in vivo and in vitro has been studied in various systems for a long time. In the current study, GPR39 activation could trigger the activation of Gαq, Gα12/13, and Gαs pathways. The activation of GPR39 by endogenous Zn ions regulates the release of Ca^2+^ from the endoplasmic reticulum (ER) through the Gαq‐PLC pathway, which further activates the MAPK and PI3K signaling pathways. In addition, the activation of the PI3K/mTOR pathway promotes the release of Zn^2+^, which further regulates the prolonged activation of MAPK and PI3K. Treatment of T84 cells with either TC‐G1008 or ZnSO_4_ remarkably increases the fluorescence ratio of Ca^2+^‐bound to Ca^2+^‐free indo‐1 (F405/F490) and significantly decreases Ca^2+^i elevation after pre‐treatment with the PLC inhibitor U73122 (10 μM) and CaMKKβ inhibitor (Figure 1). Therefore, TC‐G1008 and Zn induced Ca^2+^i elevation via the Gαq‐PLC‐CaMKKβ pathway.^30^, ^31^, ^32^, ^33^ Activation of GPR39 facilitates the reassembly of ZO‐1 into intercellular junctions in a CaMKKβ‐ and AMPK‐dependent manner, thereby improving intestinal barrier function (Figure 1).^34^These processes are essential for cell survival and proliferation. Moreover, Zn triggers the activation of ZnR/GPR39, which regulates the MAPK and PI3K pathways, thereby increasing total and phosphorylated AKT levels in PC3 cells.^13^ Silencing ZnR/GPR39 did not inhibit the upregulation of pAKT by IGF‐1. Zn^2+^ activates PI3K/AKT through ZnR/GPR39 independent of the IGF‐1R pathway. The increased Zn^2+^ dependence of pAKT was abolished upon the application of Zn^2+^ in the presence of the Gαq inhibitor YM‐25489038.^32^ In SHSY‐5Y cells and primary cortical neurons, Aβ binding to Zn in the presence or absence of Aβ does not abrogate the activation of the AKT pathway, and it is not mediated by GPR39.^35^

In the nervous system, Zn^2+^ is triggered by increases in the phosphorylation of ERK1/2 through mZnR/GPR39 in cortical neurons and the activation of mZnR/GPR39, which increased the expression of Clu.^35^ In endothelial cells, the inhibition of Gαq by YM254890 (1 μM) almost completely blocked Zn^2+^‐dependent signaling, and the PLCβ inhibitor u73122 (10 μM) mostly abolished Zn^2+^‐triggered signaling.^36^ The p‐ERK/ERK ratio in keratinocytes increased significantly from 45 to 360 min after TC‐G 1008 treatment and reached its maximum effect at 2 h. The induction effect gradually decreased after 6 h.^37^ GPR39 silencing attenuated Zn‐induced ERK1/2 phosphorylation.^38^ Similarly, Zn^2+^‐induced increases in phosphorylated ERK1/2 (pERK1/2) were reversed by Gαq inhibitors and transfection with siGPR39 constructs.^32^, ^38^ However, GPR39 activation in keratinocytes could induce ERK phosphorylation through the PI3K/MKK/MAPK pathway and not through the PLC, PKA, Ca^2+^i, and β‐arrestin pathways.^38^

Furthermore, in an ulcerative colitis model, ZnR/GPR39 could enhance the restoration of occludin expression by activating intracellular Ca^2+^ signaling, which is important for the restoration of the intestinal barrier.^39^ In addition, Pax7 activates Zac1, thereby agonizing GPR39 following the increased phosphorylation of CaMK‐II. This mechanism leads to p‐ERK1/2 dephosphorylation, which can promote the formation of type II muscle fibers.^40^ Moreover, active CaSR promotes ZnR/GPR39 surface expression through direct receptor interactions, thereby enhancing Zn^2+^‐dependent Ca^2+^ signaling.^13^ Studies on oral squamous cell carcinoma have shown that GPR39 mediates the regulation of p‐cofilin and FAK‐dependent signaling pathways to regulate the activation YAP (YAP/TAZ) primarily through the regulation of Gαq/11‐RhoA signaling but not Gα12/13 through the regulation of p‐cofilin and FAK (Figure 1).^41^

### 3.2 Regulation of GPR39 upstream signaling

Whether Zn is the only regulator of GPR39 when obestatin does not play an agonistic role remains unclear; thus, this point must be further explored. Researchers have found that GPR39 is a direct target gene of miR‐1914, which inversely regulates the expression of GPR39. The overexpression of GPR39 significantly regulated cell cycle‐related proteins such as cyclin D1 and p21 as well as apoptosis‐related proteins such as Bcl‐2 and Bax, which led to apparent increases in cyclin D1, upregulation of Bcl‐2 expression, accumulation of the G1 phase, and enhanced apoptosis (Figure 1). The downregulation of Bax anti‐apoptotic gene expression has also been reported.^42^ GPR39 induced a protective effect against the direct activation of glutamate toxicity, ER stress, and apoptotic cascade through the overexpression of BAX.^43^ In addition, the knockdown of DUXAP10, a molecular sponge that may regulate miR‐1914, inhibited GPR39 expression and PI3K/AKT signaling in MHCC‐97L cells (Figure 1). This study revealed that miR‐1914 inhibits cell growth by directly targeting the GPR39‐mediated PI3K/AKT/mTOR signaling pathway (Figure 1).^42^ Moreover, propofol protects the brain from I/R‐induced injury by modulating the MALAT1/miR‐182‐5p/TLR4 axis.^44^ NF‐κB could inhibit the expression of the GPR39 pathway in hippocampal cells through the mir‐182 pathway (Figure 1).^45^ However, the regulatory relationship between MALAT1 and GPR39 remains unknown.

Proteins that may interact with GPR39 were screened using the Y2H technique, and the protein kinase A inhibitor β (i.e., PKIB) was identified, which can synergize with GPR39 to specifically enhance RhoA‐mediated constitutive effects on SRE‐mediated transcription and cytoprotective effects but not on the Zn‐mediated activity of the GPR39 ligand. By contrast, the binding of Zn to GPR39 causes PKIB to dissociate from GPR39 (Figure 1).^14^ This finding also suggests a specific pattern of GPR39 expression.

Zn activates GPR39, thereby increasing cAMP production, which leads to the dissociation of the cAMP‐dependent protein kinase and nuclear translocation of the catalytic subunit of PKA. As previously described, Zn caused PKIB to dissociate from GPR39, thereby attenuating its constitutive activity. PKIB binds to the catalytic subunit of PKA, thereby inhibiting its activity. This mechanism leads to the recovery of AMP levels, at which point PKIB dissociates from PKA and returns to the plasma membrane to reassociate with GPR39 (Figure 1). This creates a negative feedback loop that can limit the overactivation of the Zn ligand by GPR39.^14^

Based on the abovementioned discussion, many signaling pathways are mediated by GPR39, which has a wide range of actions that affect cell proliferation, apoptosis, inflammatory response, oxidative stress, cell linkage, ion transport, and glutamate‐GABAergic interactions. GPR39 plays a role in some or all of the key membrane protein functions in the abovementioned processes. Most of these roles are beneficial for the organism. These findings indicate that GPR39 is neuroprotective and anti‐inflammatory, and it inhibits apoptosis and antioxidative stress, particularly in the nervous system. In‐depth studies of the mechanism of GPR39 may reveal GPR39 as a target for future drug treatments for neurological diseases.

## 4 GPR39 REGULATES ION HOMEOSTASIS

### 4.1 GPR39 and the KCC family

**FIGURE 2:**
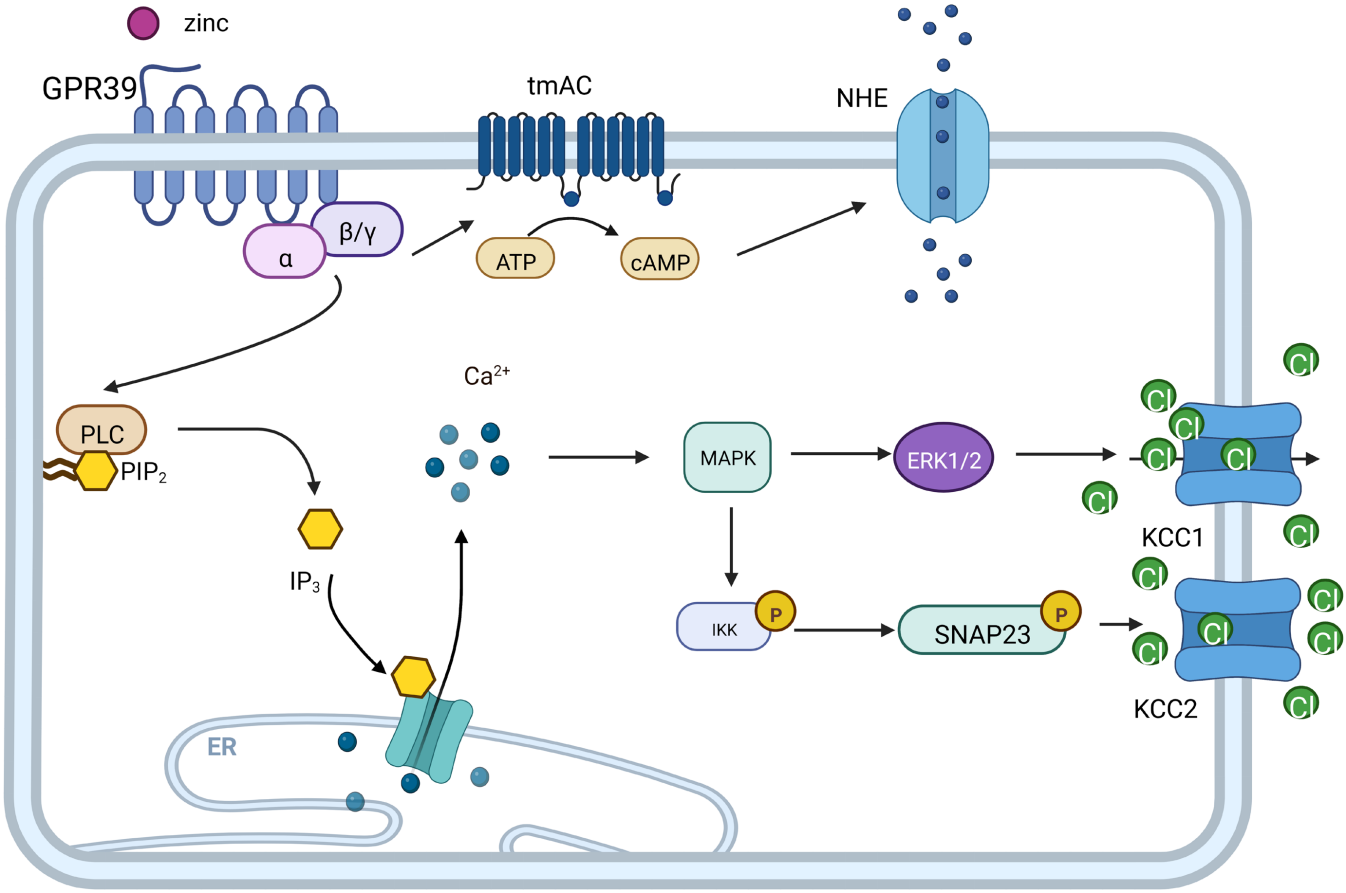
ZnR/GPR39 regulates ion transport. Agonistic GPR39 can upregulate KCC1 and KCC2 via the MAPK/ERK and MAPK/IKK pathways, respectively, which are closely related to SNAP23 phosphorylation at Ser95 and Ser120. It can also activate NHE by upregulating cAMP production through the activation of transmembrane adenylate cyclase (tmAC).

As a metabolic membrane receptor, GPR39 exhibits strong chloride regulation. The KCC family serves as the main receptor for chloride transport. Under physiological conditions, KCC co‐transport proteins constitute the major efflux pathways for K^+^ and Cl^−^, which are responsible for cell volume regulation, trans‐epithelial ion transport, and salt uptake. Zn^2+^ activates ZnR/GPR39 signaling, which upregulates KCC1 activity and enhances Cl^−^ transport, thereby reducing cholera toxin‐induced humoral accumulation. ZnR/GPR39 activation is mediated by the ERK1/2 (MAPK)‐dependent pathway to induce KCC1 upregulation (Figure 2), thereby reducing diarrheal water loss.^46^ GPR39 can also increase the expression of K^+^/Cl^−^ co‐transport protein 2 (KCC2). This protein maintains intracellular chloride concentrations, and chloride transport is critical for GABAergic inhibitory function.^8^, ^47^ The upregulation of KCC2 activity in hippocampal slices after red alginate exposure was partly due to increased surface expression of Zn^2+^‐ and mZnR/GPR39‐dependent transporter proteins. The mediated regulation of KCC2 activity requires a C‐terminal structural domain, which is achieved through the Gq‐PLC pathway.^9^, ^48^ Moreover, in neuronal SHSY‐5Y cells, Zn^2+^‐dependent KCC2 upregulation requires IKK phosphorylation signaling and SNAP23 phosphorylation at Ser95 and Ser120 (Figure 2).^48^ The expression of GPR39 also upregulated the level of KCC3, a pathway that promotes the formation of actin stress fibers, thereby promoting the release of MMP2 and MMP9. GPR39 and KCC3 regulate cell proliferation and migration.^49^ In addition, KCC3 and KCC4 mRNAs were increased in TAMR cells. Although the Zn^2+^‐dependent ZnR/GPR39 signaling pathway upregulated KCC3 activity, it did not affect KCC4. Moreover, this regulatory pathway is not dependent on Na^+^, and the use of NKCC (Na^+^‐dependent NKCC transport protein) inhibitors does not attenuate Zn^2+^‐dependent ion transport in TAMR cell translocation.^50^ KCC1 in the KCC family is considered as a housekeeper gene. Based on previous reports, KCC2, KCC3, and KCC4 play a role in Na^+^ and Cl^−^ transport in the presence or absence of K^+^.^51^ The intervention of GPR39 expression can affect the expression and function of KCC family proteins except for KCC4. Furthermore, interfering with the expression of GPR39 can affect or even become a target for treating epilepsy, depression, and diarrhea.

### 4.2 GPR39 regulates intracellular and extracellular H^+^ homeostasis

GPR39 affects the Na^+^/H^+^ exchanger NHE, a major regulator of pH. Maintaining the activity and function of neurons depends on the stability of the pH value. Thus, the change in pH can regulate the activity of nerve cells by regulating ion channel transporters. The upregulation of NHE can promote pH homeostasis.^52^ Zn activates GPR39, which then activates transmembrane adenylate cyclase to catalyze the production of cAMP. The activation of NHE (Na/H‐exchanger) is mediated by cAMP (Figure 2).^12^, ^53^ Subsequent activation of ERK1/2 enhances NHE activity and increases acidification recovery in GPR39^+/+^ neurons. Acid loading in neurons alters neuroexcitability. The mZnR/GPR39 extracellular pH (pHe)‐dependent regulation of activity does not permanently inactivate the receptor. mZnR/GPR39 is inactivated at pH values below 6.5 and above 8, and it can be reversed when cells return to physiological pHe.^53^, ^54^ Similarly, Zn^2+^ can promote epithelial repair by activating ZnR/GPR39, which activates MAP kinase and upregulates sodium/proton exchanger 1 (NHE1) activity.^55^ Limor et al. found that HT29 cells treated with Zn to activate ZnR/GPR39 also induced NHE activation, which also enhanced pH recovery in HT29 cells through Ca^2+^ signaling and ERK1/2 activation.^56^ Thus, ZnR/GPR39 plays an important role in pH homeostasis, which is essential for colon cell survival. GPR39 activation regulates NHE activity in colon cells, keratin‐forming cells, and neurons. Ensuring pH homeostasis is also critical for cell survival.

## 5 PATHOPHYSIOLOGICAL EFFECTS OF GPR39

Since the discovery of GPR39 in 1997, researchers have found that GPR39, as a “friendly” membrane protein, plays an important pathophysiological role in the human body and a regulatory role in anti‐inflammatory, antioxidant, and intercellular tight junctions.

### 5.1 GPR39 and inflammation

**FIGURE 3:**
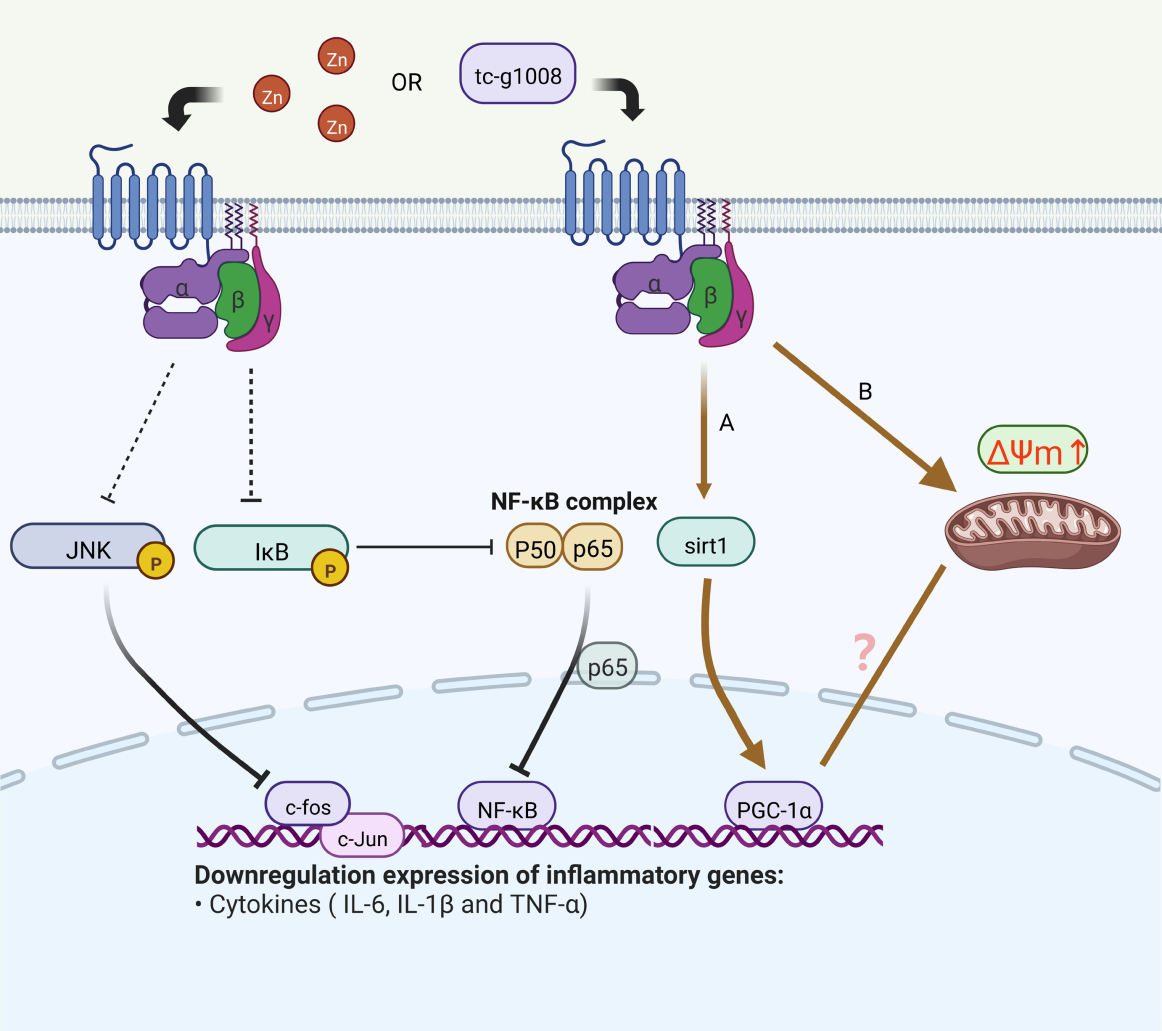
GPR39 receptor agonism (left): TC‐G1008 or Zn induces GPR39 activation, decreases IκBα phosphorylation, and reduces nuclear P65 translocation. This pathway reduces NF‐κB activation. The activation of GPR39 can also reduce the phosphorylation of JNK, leading to the reduced expression of c‐fos and c‐Jun. GPR39 receptor agonism (right): pathway A induces GPR39 activation by TC‐G1008 or Zn, which upregulates SIRT1/PGC‐1α. These pathways regulate the expression level of IL‐6, IL‐1β, and TNF‐α, thereby exerting anti‐inflammatory effects. Pathway A could exert antioxidant effects by upregulating SIRT1/PGC‐1α. The mitochondrial membrane potential was significantly increased by TC‐G1008 treatment (pathway B). Specific mechanisms underlying the antioxidant effects must be further investigated.

The stimulation of splenocytes in GPR39KO mice with lipopolysaccharide (LPS) could significantly decrease the expression level of interleukin (IL)‐1β and IL‐6.^57^ The GPR39 agonist TC‐G1008 also significantly reduced the expression level of IL‐1β and IL‐6, which was induced by TNF‐α in fibroblast‐like synoviocytes.^58^ In human aortic endothelial cells, TC‐G1008 significantly downregulated ox‐LDL‐induced mRNA levels of TNF‐α, IL‐6, and IL‐1β through GPR39 activation.^59^ Similarly, in RAW 264.7 cells, TC‐G1008 ameliorated the ox‐LDL‐induced decrease in anti‐inflammatory factor IL‐10 and elevated levels of pro‐inflammatory IL‐1β and IL‐6.^60^ Moreover, GPR39‐expressing fibroblasts can produce IL‐6 in response to injury, and Zn can promote wound healing by activating the GPR39/IL‐6 signaling axis.^61^ Apart from the abovementioned induction of pro‐inflammatory factor expression, Muneoka et al. found that TC‐G 1008 enhanced LPS‐stimulated thioglycolate‐induced production of IL‐10 by peritoneal macrophages in vitro, which exhibited evident anti‐inflammatory effects.^62^ In recent years, studies on hypoxic–ischemic encephalopathy have shown that the activation of GPR39 could downregulate IL‐6, IL‐1β, and TNF‐α expression, revealing the anti‐inflammatory function of GPR39, which indicates the potential involvement of the SIRT1/PGC‐1β/Nrf2 pathway.^11^ As a specific GPR39 agonist, the agonistic effect of TC‐G 1008 on GPR39 blocked the phosphorylation of p38 in a dose‐dependent manner. It also significantly blocked the nuclear translocation of p65 to inhibit NF‐κB pathway activation. This study revealed that GPR39 has a strong ability to regulate the activation of the p38 MAPK/NF‐κB signaling pathway (Figure 3).^33^ The activation of GPR39 significantly reduces the phosphorylation level of JNK and reduces the expression of the major components of AP‐1 (c‐fos and c‐Jun) (Figure 3).^58^ The application of TC‐G1008 reduced the phosphorylation of IκBα, thereby inhibiting the NF‐κB pathway to fight atherosclerosis (Figure 3).^59^ In addition, TC‐G 1008 (10 μM) significantly increased AMPK phosphorylation 2 h post‐treatment.^63^ Although ground‐breaking results have not been obtained on the exact anti‐inflammatory pathway of GPR39, previous studies have indicated that it may be related to the NF‐κB pathway. The activation of GPR39 can reduce the expression level of classical pro‐inflammatory factors such as TNF‐α, IL‐6, and IL‐1 β. It can also increase the level of anti‐inflammatory factors such as IL‐10. The relationship between GPR39 and inflammatory reaction is evident. Zn is a relatively “friendly” trace element, which is present at high levels in neural tissues. Thus, the anti‐inflammatory effect of GPR39, which is currently recognized as an endogenous receptor of Zn, is not difficult to understand.

### 5.2 GPR39 and oxidative stress

ZnR/GPR39 activity is present in neurons but not in glial cells.^35^ Neuronal energy production is dependent on the oxidative phosphorylation of mitochondria, and it is susceptible to oxidative stress. In addition, the mitochondrial membrane potential of HT‐22 cells treated with the GPR39 agonist TC‐G1008 was significantly increased, indicating the protective effect of GPR39 against the functional impairment of mitochondria.^5^, ^58^ TC‐G 1008 significantly downregulated ox‐LDL‐induced intercellular superoxide levels in human aortic endothelial cells, suggesting the antioxidative stress effect of GPR39.^59^ As previously mentioned, studies on hypoxic–ischemic encephalopathy found that GPR39‐mediated decreases in the expression level of IL‐6, IL‐1β, and TNF‐α promoted the anti‐inflammatory effects of these factors, which could significantly reduce the extent of cerebral ischemia caused by ischemia and hypoxia; moreover, the activation of GPR39 increased the expression level of SIRT1, PGC‐1α, and Nrf2 and reduced the number of neuronal deaths.^11^ We hypothesize that GPR39 may regulate mitochondrial membrane potential through the SIRT1/PGC‐1α pathway, which exerts antioxidant effects (Figure 3). However, this hypothesis must be further investigated, as confirmed by subsequent studies. In a recent study on osteoporosis, TC‐G1008 could accelerate reactive oxygen species (ROS) clearance by selectively activating the GPR39 signaling pathway.^64^ However, co‐treatment with Zn and Se played an important role in maintaining membrane potential levels and significantly increasing mitochondrial GPX, CAT, and MnSOD enzyme activities. This study found that GPR39 was not statistically significant in the experimental and control groups, either with a combination treatment of Zn and Se or independently.^65^ Zn does not depend on the signaling pathway induced by GPR39 to exert its effect on oxidative stress.^65^ Many studies have revealed that GPR39 inhibits antioxidant effects and ROS formation. However, significant results have not been obtained. In particular, the specific antioxidant mechanism of GPR39 in the nervous system remains unclear. Moreover, the relationship between GPR39 and mitochondria has not been reported. At present, only some factors in the oxidative stress signal pathway have been observed under ischemic and hypoxic conditions. However, the specific regulatory mechanism needs further study.

## 6 SIGNALING PATHWAYS ASSOCIATED WITH ZNR/GPR39 INDUCE MENTAL AND NEUROLOGICAL DISEASES

Given the abundant role of its endogenous ligand, such as ZnR, GPR39 plays an essential role in vivo. Results have shown that it has an irreplaceable biological role in a variety of diseases, particularly diabetes, epilepsy, depression, and cardiovascular diseases. GPR39 and its associated diseases are summarized, particularly on recent studies on neurological diseases.

### 6.1 Epilepsy: GPR39 exerts antiepileptic effects through KCC2 and affects GABAergic function

GPR39 can modulate the excitatory activity of KCC2, thereby regulating neuronal electrical activity, which is important for epilepsy control. Synaptic Zn^2+^ release in the presence of excitotoxins and subsequent mZnR/GPR39 activation could suppress the red alginate‐induced change in hippocampal electrical activity, thereby exerting a powerful antiepileptic effect. Therefore, GPR39 may serve as a therapeutic target for epilepsy.^9^ Under a deficiency in mZnR/GPR39, a single injection of erythropoietin resulted in a pronounced seizure activity.^9^ A Zn‐supplemented diet (4‐week Zn‐loaded diet) significantly improved long‐term cognitive impairment induced by developmental seizures and upregulated GPR39 expression in the hippocampus, indicating that high‐Zn intervention may induce a protective effect against developmental epilepsy‐induced brain damage via the GPR39 pathway.^66^ Recent studies have shown that in the hippocampus of GPR39‐KO mice, TC‐G 1008 treatment reduced the expression of TRPM7 and increased the expression of SLC41A1, which are important transport proteins for the movement of magnesium in and out of cells. GPR39 may also play an important role in magnesium homeostasis.^67^ Magnesium homeostasis is associated with epilepsy, and it may have synergistic effects with other antiepileptic agents.^68^, ^69^

As previously mentioned, the activation of KCC2 is closely related to intra‐ and extracellular chloride regulation and GABAergic inhibition, and the altered electrical activity of hippocampal cells is a key point for seizure suppression. Researchers may achieve seizure suppression of electrical activity by modulating the upstream and downstream signaling pathways of GPR39/KCC2. However, current research disagrees with the previous view that GPR39 activation may be a new therapeutic target, which leads to a view contrary to the previous hypothesis. In addition, zinc is a positive allosteric regulator of TC‐G1008 activity at GPR39. Dietary zinc limitation shows the inhibition of TCG‐1008 activity, whereas adequate zinc diets enhance the maximum seizure severity and percentage of completely kindled mice. Decreased dietary zinc content was associated with lower epilepsy threshold, whereas increased dietary zinc content was associated with increased epilepsy threshold.^70^ Despite the absence of a specific cellular regulatory mechanism, finding the next focus of research is not difficult, which is the root cause of this difference.

This contradictory view is more conducive to the progress of science. At present, the mainstream view is that GPR39 inhibits the occurrence of epilepsy by regulating the change in membrane potential. Critically, zinc, as an essential ingredient, may have played a crucial role. The adequacy of the zinc dose may be a significant factor affecting the severity and threshold of seizures. However, scholars still must consider whether TCG‐1008 and zinc have other regulatory pathways that affect the action of the compounds when the receptor expression of GPR39 is affected.

### 6.2 Depression: GPR39 exerts antidepressant effects by modulating the amino acid neurotransmitter system homeostasis and BDNF/TrKB signaling pathway

Despite its high prevalence, the underlying cause of depression remains unknown to date. The treatment of depression remains a serious challenge in the field of clinical medicine, and only a few patients are effectively treated. Based on the traditional monoaminergic hypothesis, the pathophysiological basis of depression is the decrease of 5‐hydroxytryptamine, norepinephrine, and/or dopamine levels in the CNS. Traditionally generated antidepressants, such as monoamine oxidase inhibitors and tricyclic antidepressants, enhance monoamine levels in synaptic spaces. Acute treatment with antidepressants, such as promethazine, escitalopram, reboxetine, and bupropion, may downregulate the expression level of GPR39 in the frontal cortex of Zn‐deficient rats. Compared with acute treatment, long‐term and sustained treatment with these antidepressants upregulated the expression level of GPR39 and the protein level of CREB, BDNF, and TrKB.^15^, ^71^, ^72^ GPR39 K0 mice are unresponsive to conventional antidepressants. Previous studies have demonstrated that GPR39 is necessary for the antidepressant effect of monoamine^71^, ^73^ and confirmed that promethazine acts through the GPR39‐dependent and ‐independent pathways.^74^ Although selective serotonin reuptake inhibitors are commonly used in antidepressant therapy in recent years, the monoaminergic system hypothesis alone cannot fully explain why most patients are ineffective.^75^

Based on the abovementioned results, the glutamatergic system appears in people's sight. By inducing KCC2 activation, GPR39 stimulates GABAergic transmission, thereby maintaining homeostasis between the GABAergic and glutamatergic systems. An imbalance between the two brain neurotransmitter systems (glutamatergic and GABAergic) may be related to the pathogenesis of depression.^47^ GPR39 agonists also have long‐lasting antidepressant effects, and research scholars have focused on this issue.^76^ Hippocampal BDNF levels were significantly increased 24 h after GPR39 agonism with a GPR39 agonist. GPR39 requires BDNF to exert its antidepressant effect. In addition, GPR39 agonists may have sedative properties, as evidenced by a considerable reduction in total arm entry in the elevated plus maze test in a previous study.^26^ Therefore, GPR39 may regulate depressive symptoms via two pathways: homeostatic alteration between GABAergic and glutamatergic modulation of mood and modulation of the BDNF/TrKB signaling pathway to exert antidepressant effects.

GPR39 not only affects the action of monoaminergic drugs but also maintains the balance of neurotransmitters in the glutamatergic system. Increased synaptic zinc release activates the upregulation of KCC2 mediated by GPR39, which provides protection against high levels of stimulation and subsequent excitotoxicity. If GPR39 is necessary to maintain the regulation of neuronal excitability, then drugs targeting GPR39 could affect two or more neurotransmitter systems simultaneously to control depression. Thus, the future of depression treatment has become clearer.

### 6.3 Dementia: GPR39 deficiency associated with cognitive impairment

Aβ plays an important role in Alzheimer's disease. The presence of Aβ plaque is the main factor affecting the differential diagnosis of diseases. Zinc is abundant in the brain. It primarily exists in the cortical amygdala and hippocampal neurons.^77^ The response of Ca^2+^i to Zn was attenuated in the presence of Aβ1‐42, and further studies revealed that Aβ attenuated Zn‐dependent attenuation of GPR39 activity.^35^ AD is associated with mitochondrial dysfunction, oxidative stress, and metal ion homeostasis. Mitochondrial SOD, GPX, and CAT activities were significantly reduced in AD rats, and Zn + Se combination treatment significantly increased the activity of these enzymes compared with the AD group. Although Zn + Se combination treatment did not affect GPR39 expression, its expression was significantly reduced in the brains of AD rats.^65^ Perivascular expression was increased in the MCI formalin‐fixed dorsolateral prefrontal cortex (dlPFC) of elderly individuals compared with that of young individuals. By contrast, microglial expression was increased in the MCI dlPFC compared with that in the older and younger controls. Although no direct evidence has shown that the GPR39 SNP is associated with cognitive impairment, pure SNPs in the GPR39‐encoding gene may accelerate the progression of white matter hyperintensities. At present, this SNP is an MRI marker for VCI. GPR39, a metabotropic zinc receptor, affects declarative memory in male mice. When GPR39 was knocked out, male mice of all ages showed amnesia. GPR39 can also regulate the expression of key elements in age‐related inhibitory neurotransmission.^78^, ^79^ In addition, a high‐fat diet and a standard diet exhibit impaired spatial memory in the absence of GPR39.^80^ Moreover, GPR39 regulates oxygen lipid synthesis, and GPR39 deletion induces the AA COX metabolites PGE2 and PGD2, 15‐keto PGF2a, and DHA 12/15‐LOX metabolite resolvin D1, indicating that constitutive GPR39 activity suppresses COX and 12/15‐LOX. These oxygenated lipid synthases and GPR39 may serve as biomarkers or targets for VCI therapy.^80^

At present, many studies have reported on the relationship between zinc and dementia, but there are still controversies. Although most studies suggest that zinc is beneficial, some scholars still believe that zinc has neurotoxicity, which may be explained by the different binding sites between different concentrations of zinc and APP. In addition, no clear causal relationship is found between the transmission of zinc neurotransmitters mediated by GPR39 and memory. GPR39 is expressed in the dorsolateral prefrontal cortex of the elderly. Immunohistochemical analysis showed that it primarily existed in pericytes and microglia.^78^ With age, the increase in inflammatory factors and ROS caused by the aggravation of hypoxia in the whole brain can damage the blood–brain barrier and cause white matter damage. Finally, neurodegenerative diseases can also occur. This can also be a direction for GPR39 to intervene in the diagnosis and treatment of dementia.

### 6.4 Stroke: GPR39 may be a future therapeutic target

A recent study on stroke found a negative association between low Zn levels and ischemic stroke (IS), that is, low Zn levels may be a risk factor for IS.^81^ Thus, Zn supplementation could reduce the risk of stroke or induce a protective effect. Zinc, as a neurotransmitter or neuromodulator, plays an important role in the growth and development of the brain. At present, many studies have confirmed that zinc not only has neurotoxicity but also has neurorepair and neuroprotective effects.^82^ As a specific zinc receptor, the role and mechanism of GPR39 in IS are limited. Recent studies have shown that the deletion of the GPR39 gene aggravates ischemic brain injury, particularly the reduction in capillary reperfusion, thereby aggravating ischemic brain injury. Notably, ischemic brain injury in males after GPR39KO is more severe than that in females.^83^ In addition, some scholars have found that TC‐G1008 is considered as a specific agonist of GPR39, and the ischemic area of HIE mice treated with TC‐G1008 decreased significantly.^11^ Atherosclerosis primarily causes IS. Changes in endothelial cell activity may accelerate the progression of atherosclerosis. Based on previous reports, GPR39 is closely related to vascular endothelial cells. Extracellular Zn^2+^ can regulate not only microvascular tension by enhancing endogenous arachidonic acid 15‐HETE and 1415‐EET but also endothelial cell activity by activating the downstream Gαq‐PLC pathway induced by GPR39. It also promoted vascular cell survival and growth by increasing the expression level of cAMP and Akt and by overexpressing platelet‐derived growth factor‐α receptor and vascular endothelial growth factor A.^36^, ^84^ Moreover, zinc has a potentially strong inhibitory effect on vascular calcification by partially regulating the GPR39‐mediated upregulation of TNFAIP3.^85^, ^86^ The increase in GPR39 expression not only reduces the number of apoptotic macrophages but also inhibits the lipid accumulation of macrophages induced by ox‐LDL to slow down the progression of atherosclerosis.^60^ The debate on metal homeostasis has become increasingly intense in recent years, and it has been suggested that disturbances in metal homeostasis are important factors affecting the pathogenesis of androgen insensitivity syndrome.^87^ A 2019 scholarly study of 1277 patients newly diagnosed with stroke found that high plasma concentrations of Al and Cd and low concentrations of iron and selenium may increase the risk of IS.^88^ A recent meta‐analysis showed that higher Zn levels may be associated with an increased risk of IS.^89^

Therefore, maintaining Zn homeostasis is crucial.^6^ Based on the present findings, Zn has two sides, particularly in IS. However, the specific relationship between GPR39, as a zinc receptor, and stroke must be further studied. Stroke results from a series of complex pathophysiological changes, which involve many pathophysiological mechanisms such as calcium flow, oxidative stress, and inflammation. Evidently, GPR39 plays a regulatory role in such mechanisms. Thus, our experimental group aims to understand the specific mechanism of GPR39 in IS in the future. Classical molecular biological mechanisms after stroke include calcium overload of nerve cells, excitatory amino acid toxicity, reperfusion injury, and inflammatory cell apoptosis. Determining whether GPR39 is closely related to the abovementioned mechanisms is not difficult; thus, GPR39 is likely to be an important therapeutic target for stroke.

## 7 GPR39 AGONISTS

In 2016, an in‐depth study of GPR39 agonists identified several novel GPR39 agonists, such as the JAK2 inhibitor LY2784544 and the PI3Kb inhibitor GSK2636771. This study also revealed the role of Zn^2+^ among the three agonists, with Zn acting as a positive metastable modulator (PAM) of GPR39‐C3 activity with regard to efficacy, whereas Zn^2+^ positively regulated PAM efficacy and LY2784544 and GSK2636771 activity potency.^90^ In addition, three GPR39 agonists, namely, AZ7914, AZ1395, and AZ4237, did not promote hypoglycemia; these agonists also exhibited proinsulin effects in rodents.^91^ This finding was followed by the discovery of a novel agonist of GPR39, namely, Cpd1324, which served as a weight‐loss drug when taken orally and regulate the secretion of glucagon‐like peptide‐1 (GLP‐1) by activating the Ga‐subunit.^92^ The exploration of GPR39 agonists has not stopped. In 2017, a study showed that GSB‐118 served as a biased ligand for GPR39 and enhanced the ZnCl2‐induced cAMP reaction but without any agonist activity or effect on the Ca^2+^ reaction.^93^ Frimurer et al. also proposed that Zn could serve as a metastable enhancer of GPR39 and viewed the novel compound as an agonist. The combination of the two not only changes the binding site of GPR39 to Zn but may also generate new biological effects.^94^ The current unified opinion is the selective agonistic effect of TC‐G1008 on GPR39. In exploring the role of GPR39, researchers have conducted structural studies on the agonists of GPR39 and found that 2‐pyridylpyrimidines have the most active oral bioavailability and may strongly induce GLP‐1 expression (approximately sixfold),^95^ which is ~10 times more effective than ZnSO_4_ in increasing TEER.^62^ As a calcium (Ca^2+^)‐sensing protein located in the ER membrane, STIM2 plays an important role in Ca^2+^ homeostasis. The expression of GPR39 was found to be upregulated in stim2b^−/−^ pups. Whether STIM2 plays a role in the suppression of GPR39 expression requires further investigation.^96^ Although most studies have not accounted for them separately, they have used siRNA‐mediated GPR39 knockdown to determine whether the same can be achieved using specific inhibitors. In vivo conditions used to treat diseases are easier to control, particularly for tumors in which GPR39 inhibition can inhibit cell proliferation; thus, such work may be the next step for further exploration.

## 8 PROSPECT OF GPR39 IN DISEASE AND HEALTH

Over the years, scholars have devoted themselves to the pathophysiology of GPR39 in various systems. However, there are still some problems that need to be discussed in depth. Previous studies have found that GPR39 activation have specific effects on many diseases. It also showed a friendly response to diseases, particularly diabetes, heart disease, osteoporosis, tumor, and enteritis. For example, GPR39 knockout increases blood glucose level and decreases insulin secretion.^97^, ^98^ Although GPR39 can affect insulin secretion, the exact direct mechanism remains unclear. The specific mechanism underlying GPR39‐induced GLP‐1 and GIP secretion was not explored in the current study. Second, GPR39 mediates cardiac hypertrophy through the MTOR/S6K1 pathway mediated by AMPK.^99^ In particular, GPR39 regulates vascular endothelial cell activity and vascular calcification, and it has anti‐atherosclerosis effect. A new field of GPR39 research has also emerged. The results of cardiovascular disease research in recent years are very exciting. Although GPR39 can promote the proliferation of intestinal cells, protect the functional stability of intestinal epithelial cells,^39^, ^100^ and promote the proliferation of tumor cells through the PI3K/MAPK pathway and upregulation of cyclin,^13^, ^32^, ^101^ it can evidently reduce cell proliferation after being inhibited.^102^ The emergence of inhibitors may also provide a new idea for tumor‐targeted therapy. At present, the specific inhibitor of GPR39 has not been reported. Thus, the emergence of GPR39 inhibitors will make a breakthrough.

Some studies on nervous system diseases have been reviewed and discussed in detail in this paper. However, there are still some issues that need to be discussed. First, only phenomenal studies on stroke have been published. In‐depth mechanism research and discussion are also lacking. Combined with cardiovascular studies, GPR39 has been found to affect endothelial cell activity and vascular calcification. Moreover, the HIE model, which is similar to the MCAO model, found that GPR39 has neuroprotective, anti‐inflammatory, and antioxidant effects.^11^ Multiple sclerosis is an autoimmune disease with relapse and remission. Some cases may also show irreversible neurodegeneration, and inflammatory change is its core pathological change.^103^ With regard to the mechanism, GPR39 may improve the pathogenesis of multiple sclerosis, which remains unclear and needs further research. Similar to multiple sclerosis, few studies have been conducted on neurodegenerative diseases related to dementia. Its high expression in the hippocampus and amygdala reveals its close relationship with cognition from the side.

Zn supplementation has become an effective but inappropriate therapeutic strategy because of its multiple functions in vivo and possible neurotoxicity. The synthetic agonist of GPR39 makes up for this shortcoming. TC‐G1008 is prominent in many systemic diseases, particularly in nervous system diseases. Therefore, the improvement of synthetic agonists will promote the progress of GPR39 research. In particular, the bioligand theory discovered in recent years suggests that compounds can enhance the effect of Zn and change the binding site between GPR39 and Zn to produce new biological effects. This finding may be the focus of future research.

## 9 CONCLUSION

ZnR/GPR39, as a member of the G protein‐coupled family, plays various important biological roles in vivo, including the promotion of anti‐inflammatory and antioxidant activity, inhibition of apoptosis, promotion of cell proliferation, maintenance of cell linkage stability, and regulation of ion homeostasis. In recent years, Zn has been identified as an endogenous ligand of GPR39, which accelerates the research progress of the pathophysiological function of GPR39.^90^, ^91^, ^92^, ^93^ Most studies have focused on the role of GPR39 in diabetes, psychiatric disorders, and intestinal diseases. However, few studies have been conducted on its role in neurological disorders. Published papers have clearly demonstrated that GPR39 has positive neuroprotective effects as a membrane protein. The innovation of agonists will promote the research progress of GPR39 in the nervous system. It activates a powerful neuroprotective effect through its unique signal pathway. In the near future, GPR39 can be a target in neurological diseases for targeted therapy, which will help doctors overcome the associated problems. In addition, the discovery of synthetic agonists such as TC‐G1008 has expanded researchers' thinking. The current views on the controversial obestatin are not consistent.^104^, ^105^ Therefore, we are looking forward to the new GPR39 agonist discovered recently. In the next few years, the understanding of the physiological function of GPR39 will deepen with the emergence of GPR39 agonists.

## AUTHOR CONTRIBUTIONS

CB designed the review paper and wrote the manuscript. WJ and FJ reviewed and revised the final version of the manuscript and supervised the study.

## FUNDING INFORMATION

This work was supported by the National Natural Science Foundation of China (grant number 82271353 to JW and grant number 82271275 to JF) and the Outstanding Scientific Fund of Shengjing Hospital (to JF).

## CONFLICT OF INTEREST STATEMENT

The authors declare that they have no competing interests.

## Data Availability

Data sharing not applicable to this article as no datasets were generated or analyzed during the current study.
